# Comparative Genomics of 2009 Seasonal Plague (*Yersinia pestis*) in New Mexico

**DOI:** 10.1371/journal.pone.0031604

**Published:** 2012-02-16

**Authors:** Henry S. Gibbons, Michael D. Krepps, Gary Ouellette, Mark Karavis, Lisa Onischuk, Pascale Leonard, Stacey Broomall, Todd Sickler, Janet L. Betters, Paul McGregor, Greg Donarum, Alvin Liem, Ed Fochler, Lauren McNew, C. Nicole Rosenzweig, Evan Skowronski

**Affiliations:** 1 United States Army Edgewood Chemical Biological Center, Aberdeen Proving Ground, Maryland, United States of America; 2 Science Applications International Corporation, Aberdeen Proving Ground, Maryland, United States of America; 3 New Mexico Department of Public Health, Albuquerque, New Mexico, United States of America; 4 OptiMetrics, Inc., Abingdon, Maryland, United States of America; 5 Excet, Inc., Springfield, Virginia, United States of America; Argonne National Laboratory, United States of America

## Abstract

Plague disease caused by the Gram-negative bacterium *Yersinia pestis* routinely affects animals and occasionally humans, in the western United States. The strains native to the North American continent are thought to be derived from a single introduction in the late 19^th^ century. The degree to which these isolates have diverged genetically since their introduction is not clear, and new genomic markers to assay the diversity of North American plague are highly desired. To assay genetic diversity of plague isolates within confined geographic areas, draft genome sequences were generated by 454 pyrosequencing from nine environmental and clinical plague isolates. *In silico* assemblies of Variable Number Tandem Repeat (VNTR) loci were compared to laboratory-generated profiles for seven markers. High-confidence SNPs and small Insertion/Deletions (Indels) were compared to previously sequenced *Y. pestis* isolates. The resulting panel of mutations allowed clustering of the strains and tracing of the most likely evolutionary trajectory of the plague strains. The sequences also allowed the identification of new putative SNPs that differentiate the 2009 isolates from previously sequenced plague strains and from each other. In addition, new insertion points for the abundant insertion sequences (IS) of *Y. pestis* are present that allow additional discrimination of strains; several of these new insertions potentially inactivate genes implicated in virulence. These sequences enable whole-genome phylogenetic analysis and allow the unbiased comparison of closely related isolates of a genetically monomorphic pathogen.

## Introduction

*Yersinia pestis*, the etiologic agent of plague disease, exists worldwide in discrete enzootic foci that contain genotypically and often phenotypically distinct variants [1], [2]. Three major waves of plague have spread worldwide due to human migrations and international trade [3], the third and most recent of which occurred in the late 19^th^ and early 20^th^ centuries. The third pandemic resulted in the establishment of new plague-endemic regions in North and South America, where it was previously not documented. Each of the plague pandemics are now thought to have been caused by a nitrate-reducing, glycerol-non-fermenting biotype known commonly as the Orientalis biotype [4], [5], [6]. However, some recent reports, including a whole-genome sequence of an isolate from the 14^th^ century pandemic, demonstrate that strains from a more deeply rooted branch of the *Y. pestis* lineage may have caused the Black Death [7], [8]. Phylogenetic analysis based on VNTRs established the phylotype of the North American isolates as 1.ORI1 [9]. Because no pre-existing plague foci were present on the North American continent, the plague reservoirs (mostly in small rodent populations) contained exclusively this biotype and are thought to have relatively little genetic diversity. However, the apparent lack of diversity may be the result of discovery bias, as the phylogenetic analyses to date have relied on only three North American plague genome sequences [1], [10], [11], [12].

In New Mexico, plague season begins in May and ends in September, although plague occurs year round. Based on data collected by the New Mexico Department of Health's Epidemiology and Response Division (ERD) (http://www.health.state.nm.us/erd/HealthData/plague.shtml) there were an unusually high number of human cases between 1975 and 1985, including several deaths, whereas pet cases did not appear to follow a similar trend. The majority of human cases are clustered in the North Central region of New Mexico and population density does not appear to be a factor; Bernalillo, Sandoval, and Santa Fe counties have high population densities and high case loads, but Rio Arriba and McKinley counties have medium to low population densities with high case loads. Pet cases appear to be clustered in Bernalillo (Albuquerque) and Santa Fe counties. Data are not available for the distribution of plague in surveillance animals (rodents and lagomorphs).

While the genetic diversity of North American plague strains is thought to be limited, few whole-genome sequences (WGS) are available for strains originating on this continent. These sequences have shown some degree of variation that is suggestive of the presence of genetic diversity at the nucleotide level [10], [11]. Furthermore, the extraordinary resolution of modern whole-genome sequencing allows the investigation of phylogeny at unprecedented breadth and depth. Unlike multilocus sequence typing (MLST) or multilocus variable-number tandem repeat analysis (MLVA), the most broadly used typing schemes to date [9], [13], WGS provides a complete inventory of the nucleotide sequence of an organism and allows a direct comparison of the coding capacity of a new strain to that of a reference sequence.

In cases where deliberate use of a biological agent is suspected, WGS is expected to play a critical role in identifying the origin of the outbreak strain and establishing epidemiological connections between the attack materials and potential sources, as exemplified by the investigation of the 2001 Anthrax attacks [14]. The ability to establish forensically defensible genetic links between outbreak strains and the large potential genetic reservoir of laboratory-cultured and natural isolates requires the availability of a large sample of WGS derived from genetically diverse collections with well-established geographic and phenotypic correlates.

In this report we present a focused investigation of a representative sample of plague cases originating in Northern New Mexico. We combine traditional phylogenetic typing techniques with whole-genome sequencing to correlate geographic separation with phylogenetic divergence, dramatically expanding the number of available SNPs and structural variations for phylogenetic and bioforensic discrimination of North American plague strains.

## Methods

### Sample collection

The New Mexico Department of Health, Scientific Laboratory Division (SLD) has a close working relationship with the state Office of the Medical Investigator (OMI), and the NM Department of Agriculture Veterinary Diagnostic Services (VDS). SLD performs routine bacteriological testing for OMI, VDS, and for clinical laboratories throughout the state. Additionally, SLD is the only reference lab for select agents in New Mexico, and as such, all select agents are confirmed there. Specimens from presumptive plague cases could be submitted for identification via serology or via bacterial isolation; the specimens collected in this study were a representative sample of those submitted for bacterial isolation. Isolates or specimens from clinical or veterinary sources were sent to SLD from OMI as routine bacterial cultures; from clinical laboratories for exclusion testing; or from a variety of sources including blood, lymph node aspirates and tissue samples. Animal specimens submitted through VDS were received through surveillance testing (rodents and lagomorphs) or from veterinary clinics (pets). The Department of Health's Zoonoses team conducts routine trapping of rodents in known plague enzootic areas. Rodent die-offs (e.g., a previously active prairie dog colony that has suddenly disappeared and/or single sightings [rabbit carcasses]) are usually reported by individuals to the Epidemiology and Response Division or Albuquerque Environmental Health Department's BioDisease Management Program. They, in turn, actively sample any available material from such areas and submit the carcasses for further investigation to the VDS, which is co-located with SLD. Veterinarians also send specimens collected from pets to VDS after which SLD performs the testing.

### Laboratory identification

Clinical specimens or bacterial isolates were plated onto blood agar at 25°C and 35°C and chocolate agar plates at 35°C and incubated up to one week. Presumptive plague isolates were identified by inspection of colony and microscopic morphology, growth characteristics, direct fluorescent antibody staining, specific bacteriophage lysis, and when applicable, production of catalase and oxidase. Laboratory-confirmed isolates were transferred to the Edgewood Chemical Biological Center (ECBC, Maryland, USA) under established regulations and procedures for Select Agent transportation (see Supplementary Text for details).

### Molecular methods

*Yersinia pestis* agar slants received from the New Mexico Department of Health were used to streak Tryptic Soy Agar (TSA) plates (Culture Media and Supplies) in order to obtain single isolate colonies. These plates were then placed at 30°C and incubated for 48 hours. After incubation, a single colony was picked from the TSA plates and used to inoculate 12 ml of Tryptic Soy Broth (Remel) in sterile TPP 50 ml TubeSpin Bioreactor conical tubes. To avoid plasmid loss and other artifacts of *in vitro* cultivation, strains were minimally passaged prior to sequencing. The cultures were incubated at 30°C while shaking at 150 RPM for 48 hours or until saturated growth was achieved. Genomic DNA was then isolated using the MoBIO UltraClean Microbial DNA Isolation Kit (MoBIO).

### Whole-genome shotgun sequencing and assembly

WGS was performed on the Roche/454 GS-FLX Titanium sequencing platform according to standard methods. Reads were assembled using Newbler 2.3 in an automated pipeline combining *de novo* and template-assisted assembly methods (N. Rosenzweig *et al.*, manuscript in preparation). Reads from each dataset were mapped to the published sequence of the CO92 reference strain [12] using GSMapper v2.3. Sequencing and mapping statistics are available in Supporting Information File S1.

### Identification of SNPs

Reference-based assemblies were interrogated for high-confidence differences, which were identified from the Newbler HCDiffs.txt output files (See Supporting Information File S2) in well-covered regions of the genome (>5 reads, 85% divergence from the reference, high quality scores of the mutated base and surrounding bases) that were free of potential assembly conflicts. These were considered to be potential SNPs relative to the reference sequence. Potential errors in the reference sequence were identified by performing a reference assembly with a 454 dataset from DNA isolated from a *Y. pestis* CO92 strain in our inventory. Putative SNPs were screened by BLAST against previously published *Y. pestis* sequences in GenBank to identify novel sequence variants. Potential variants that matched multiple regions on the CO92 genome were discarded as repetitive regions. In one case, a homopolymer tract was formed as the result of a deletion (at CO92 position 2238568); in that case, all assemblies were manually inspected to verify the deletion. The effects of the SNPs on putative protein sequences were analyzed using LaserGene (DNASTAR, Madison WI). NCBI accession numbers for the WGS data are given in Table 1.

**Table 1:** Strains and origins.

Table 1: 2009 New Mexico Plague Isolate Data							
Map	Strain ID	DateIsolated	Host	Tissue	Location	Reference	Accession Number
a	CO92	1992	Human	Unknown	Colorado	[33]	
3	BA200901799	6/17/2009	Human female	Blood	Santa Fe, NM	This Study	AGJS00000000
7	AS200901539	7/9/2009	*Cynomys gunnisoni*	Liver/spleen	Las Vegas, NM	This Study	AGJT00000000
1	AS200901156	6/10/2009	Feline Female, Age 12	Blood	Santa Fe, NM	This Study	AGJU00000000
2	BA200901703	6/10/2009	Human male	Groin aspirate	Santa Fe, NM	This Study	AGJV00000000
6	BA200901990	7/8/2009	Human female	Blood	Edgewood, NM	This Study	AGJW00000000
8	BA200902009	7/10/2009	Human Male	-	Edgewood, NM	This Study	AGJX00000000
9	AS200902147	8/14/2009	*Cynomys gunnisoni*	Liver/spleen	Santa Fe Airport	This Study	AGJY00000000
4	AS200901434	6/30/2009	*Sylvilagus audobonii*	Liver/spleen	Santa Fe, NM	This Study	AGJZ00000000
5	AS200901509	7/7/2009	*Cynomys gunnisoni*	Liver/spleen	Santa Fe, NM	This Study	AGKA00000000

### Identification of IS element insertion points

High-confidence structural variants were identified in the reference-based assemblies using the 454HCStructVars.txt file in the Newbler output (See Supporting Information File S3). Reads spanning the junction points were identified in the assemblies, and the unmapped or chimeric portion was used to query the NR database in GenBank. IS element insertions were assigned when the BLAST results from reads on both 5′ and 3′ ends of a potential insertion matched the termini of a single common IS element (For example see Figure S2).

### MLVA analysis

MLVA loci were amplified by PCR and their lengths determined empirically by capillary electrophoresis according to the method of LeFleche *et al.* [15]. The primer sequences used to amplify the MLVA loci were also used to query the *de novo* WGS assemblies and published sequence data, and thereby determine the lengths of the *in silico* assembled loci using LaserGene v8.1.

### Phylogenetic analysis (MLVA)

Phylogenetic relationships were constructed under the Maximum Parsimony (MP) and Neighbor Joining (NJ) methods implemented in PAUP 4.0b10 (Swofford, 1999). MP analysis with equally weighted characters were computed via a full heuristic search using tree bisection-reconnection branch swapping with random taxon addition replicates (minimally 1000) to reduce the chance of missing the most optimal solution due to being isolated within a tree island. Outgroup comparison was conducted using *Y. pestis* Pestoides F [16]. Six characters were parsimonious informative; tree reconstruction found 273 most parsimonious trees with Tree length = 19 (Consistency index (CI) = 0.842, Retention index (RI) = 0.786, Rescaled consistency index (RC) = 0.662). Clade support was assessed using the non-parametric bootstrap [17] under the same search conditions described above for MP (1000 replicates). The NJ analysis was computed using the mean character difference distance measure (tree score = 2.07892) with clade support assessed by bootstrap analysis.

### Phylogenetic analysis (SNP and IS elements)

Phylogenetic analyses were carried out using both ML and MP methods.

#### ML method

The evolutionary history was inferred by using the Maximum Likelihood method based on the Tamura-Nei model [17]. Initial tree(s) for the heuristic search were obtained automatically as follows. When the number of common sites was <100 or less than one fourth of the total number of sites, the MP method was used; otherwise BIONJ method with MCL distance matrix was used. The tree is drawn to scale, with branch lengths measured in the number of substitutions per site.

#### MP method

The bootstrap consensus tree inferred from 1000 replicates is taken to represent the evolutionary history of the taxa analyzed [17]. Branches corresponding to partitions reproduced in less than 50% bootstrap replicates are collapsed. The MP tree was obtained using the Close-Neighbor-Interchange algorithm (pg. 128 in ref. [18]) with search level 1 in which the initial trees were obtained with the random addition of sequences (10 replicates). Both ML and MP analyses utilized nucleotide sequences from 13 strains. All positions containing gaps and missing data were eliminated. There were a total of 79 positions in the final dataset; this number includes SNPs named in this research as well as positions that were added to include the outgroup and positions published previously in the CA-88/FV1 work. Evolutionary analyses were conducted in MEGA5 [19].

## Results

### Collection and genomic analysis

**Figure 1:**
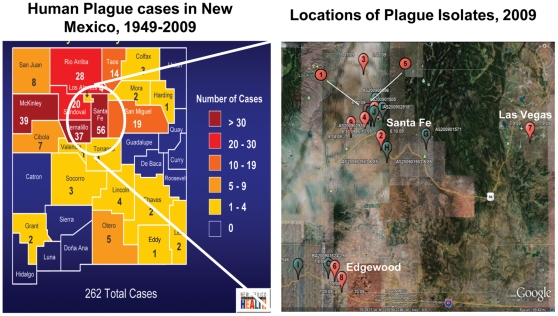
New Mexico is a Portion of the Enduring North American Plague Focus. Highly virulent *Yersinia pestis* can be found among numerous rodent and animal hosts, occasionally infecting humans and their pets when they come into contact with infected animals or fleas. Samples were chosen for sequencing to represent examples coming from each broad geographic region. Common hosts include squirrels, cats, prairie dogs (*Cynomys gunnissoni*) and rabbits (*Sylvilagus audubonii*) [3]. Sequenced isolates are shown with red markers; other cases with green markers (Location map on right). Locations of isolation of strains were mapped using Google Earth®. Source: New Mexico State Dept. of Public Health [32].

Ongoing biosurveillance efforts carried out by the New Mexico State Department of Public Health resulted in the acquisition of 15 *Y. pestis* isolates from animal and patient samples. Nine strains that constituted a geographically representative sampling of isolates were sent to ECBC for sequencing (Table 1, Figure 1). These strains were distributed across three counties in northern New Mexico. In addition, a comparable 454 dataset from CO92 was also generated. WGS statistics are presented in Supporting Information File S1. The reads from each dataset were mapped to the previously published *Y. pestis* CO92 chromosome and plasmid sequences [12]. The raw high-confidence differences derived from the HCDiffs.txt files are given in Supporting Information File S2. All mutations identified as Indels were manually inspected to confirm that they did not fall in homopolymer regions (Figure S1, S2).

### MLVA analysis

*In vitro* and *in silico* MLVA results were generally comparable (Supporting Information File S1). *In silico* MLVA results for the well-characterized CO92 resequencing dataset matched both *in vitro* determined MLVA profiles and previously published results [9], [13], [15], suggesting that *in silico* MLVA will be applicable to multiple WGS datasets provided that sufficient coverage is obtained. In some cases, low coverage of the MLVA loci resulted in low assembly quality and consequent breaks in the assembly, particularly for the longer repeat regions. Only six out of 70 MLVA loci sequenced for this dataset failed to assemble *in silico*, and all such assembly failures occurred for VNTR amplicons larger than 200 bases. For the shorter MLVA amplicons, all assembled successfully.

The MLVA profiles of the strains were similar but not identical, with the most variation observed for the Ms62 and Ms06 loci, where six and three distinct genotypes were observed, respectively. However, none of the strains entirely matched the CO92 genotype. These results are consistent with the known variability of the MLVA loci from strains isolated within confined geographic regions [13], [20].

### Genetic diversity of strains isolated in 2009

All strains closely resembled the CO92 reference strain, with no unexpected genetic material present in any of the strains, and no large deletions were noted. One of the strains appeared to have lost the pCD1 virulence plasmid, which can be lost upon *in vitro* culture [21], but is essential for virulence; thus it is most likely that this strain lost the pCD1 plasmid after its isolation. Slight variation in the percentage of reads mapping to the pPCP1 plasmid were noted. Three strains (BA200901156, BA200901990, and BA200902009) possessed approximately half the relative plasmid copy number of the other strains in the dataset (Supporting Information File S1).

**Figure 2:**
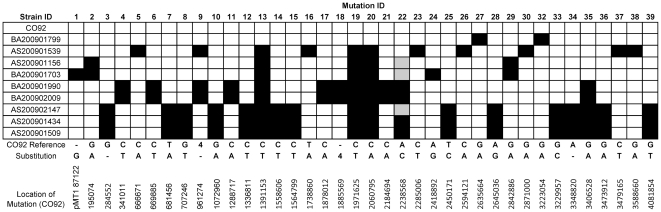
Genetic Diversity of the 2009 Plague Isolates. Distribution of mutations through the 2009 isolates. Mutations relative to the parent strain (CO92) are indicated by black squares. Grey squares indicate that the mutation was not called automatically but was evident by manual inspection of the assembly. Mutation 31 from Table 2 is not shown nor incorporated into the phylogeny as it is an expansion of a 10 bp repeating sequence.

In spite of the relatively low genetic diversity of these strains, across the entire panel of strains we found 39 total variations relative to the CO92 genome that had previously not been identified. At least two strains in the dataset contained the wild-type locus for any given mutation. In addition, the sequences were compared *in silico* to two other North American strains, FV-1 and CA88. The location of each mutation on the CO92 chromosome is presented in Figure 2, and the effects of the mutation on putative proteins are listed in Table 2.

**Table 2:** Mutations Identified in New Mexico *Y. pestis* Strains.

Replicon	Muta-tion ID #	CO92 Coor-dinates	Base(CO92/NM)	Gene(s) Affected	Annotation	Effect on Protein Sequence
pMT1	1	87122	-/G	Integrase		
Chrom	2	195074	G/A	Intergenic	YPO0174-*crp*	
Chrom	3	284552	G/-	YPO0283	*hmuR* - hemin receptor precursor	Frameshift (157/657)
Chrom	4	341011	C/T	Intergenic	YPO0331- *rhaS*, possible promoter region	
Chrom	5	666671	C/A	Intergenic		
Chrom	6	669885	C/T	YPO0610	Hypothetical protein, weak homology to glycosylhydrolases	H606Y
Chrom	7	681456	T/A	YPO0618	Hypothetical transmembrane protein	S84C
Chrom	8	707246	G/T	Intergenic		
Chrom	9	961274	4 bp del.	Intergenic		
Chrom	10	1072960	G/A	YPO0969	Hypothetical protein	A551V
Chrom	11	1288717	C/A	YPO1142	*modF* - putative molybdenum transport protein ATPase	Truncation (160/496)
Chrom	12	1336811	C/T	YPO1187	Putative substrate binding periplasmic protein	Synonymous
Chrom	13	1391153	C/T	Intergenic		
Chrom	14	1558606	C/T	YPO1383	*pfl* - pyruvate formate acetyltransferase	A578T
Chrom	15	1564799	C/T	YPO1386	*ansB* - L-asparaginase II	A313V
Chrom	16	1738860	T/A	YPO1528	*fhuF* - Ferric iron reductase protein FhuF	Synonymous
Chrom	17	1878012	C/A	YPO1652	*ylaC* - Hyopthetical protein	Q113H
Chrom	18	1885569	4 bp ins.	YPO1657	*cheM*, repeat expansion	
Chrom	19	1971625	C/T	Intergenic		
Chrom	20	2060795	C/A	YPO1813	Putative sugar binding periplasmic protein	A318E
Chrom	21	2184694	C/A	YPO1926	Putative 4-hydroxybutyrate coenzyme A transferase	Synonymous
Chrom	22	2238568	A/C	Intergenic		
Chrom	23	2285006	C/T	YPO2013	*prsA* - Ribose phosphate phosphokinase	G255E
Chrom	24	2418892	A/G	YPO2149	Hyopthetical	Synonymous
Chrom	25	2450171	T/C	Intergenic	Near IS element	
Chrom	26	2594121	C/A	YPO2306	Putative amino acid transporter	P267T
Chrom	27	2635664	G/A	YPO2341	Putative mandelate racemase/muconate	Synonymous
Chrom	28	2645036	A/G	Intergenic		
Chrom	29	2842886	G/A	YPO2553	*edd* - phosphogluconate dehydratase	R402C
Chrom	30	2871000	G/A	Intergenic		
Chrom	31	2916182	12 bp ins	Intergenic	Repeat expansion	
Chrom	32	3224054	G/A	YPO2886	*yapA* - putative autotransporter protein	Synonymous
Chrom	33	3229957	G/C	YPO2887	*yapB* (pseudogene in CO92)	Synonymous
Chrom	34	3348820	A/-	YPO2998	Putative 2-component system response regulator	Frameshift (24/227)
Chrom	35	3406528	G/A	YPO3049	Putative binding protein-dependent transport system	A564V
Chrom	36	3473912	G/A	Intergenic		
Chrom	37	3479165	C/T	YPO3112	*dnaX* - DNA pol III subunits gamma & tau	D589N
Chrom	38	3588660	G/A	YPO3224	*frsA* - fermentation/respiration switch protein	Synonymous
Chrom	39	4081854	G/T	YPO3661	Putative sulfite oxidase subunit YedZ	H164N

**Figure 3:**
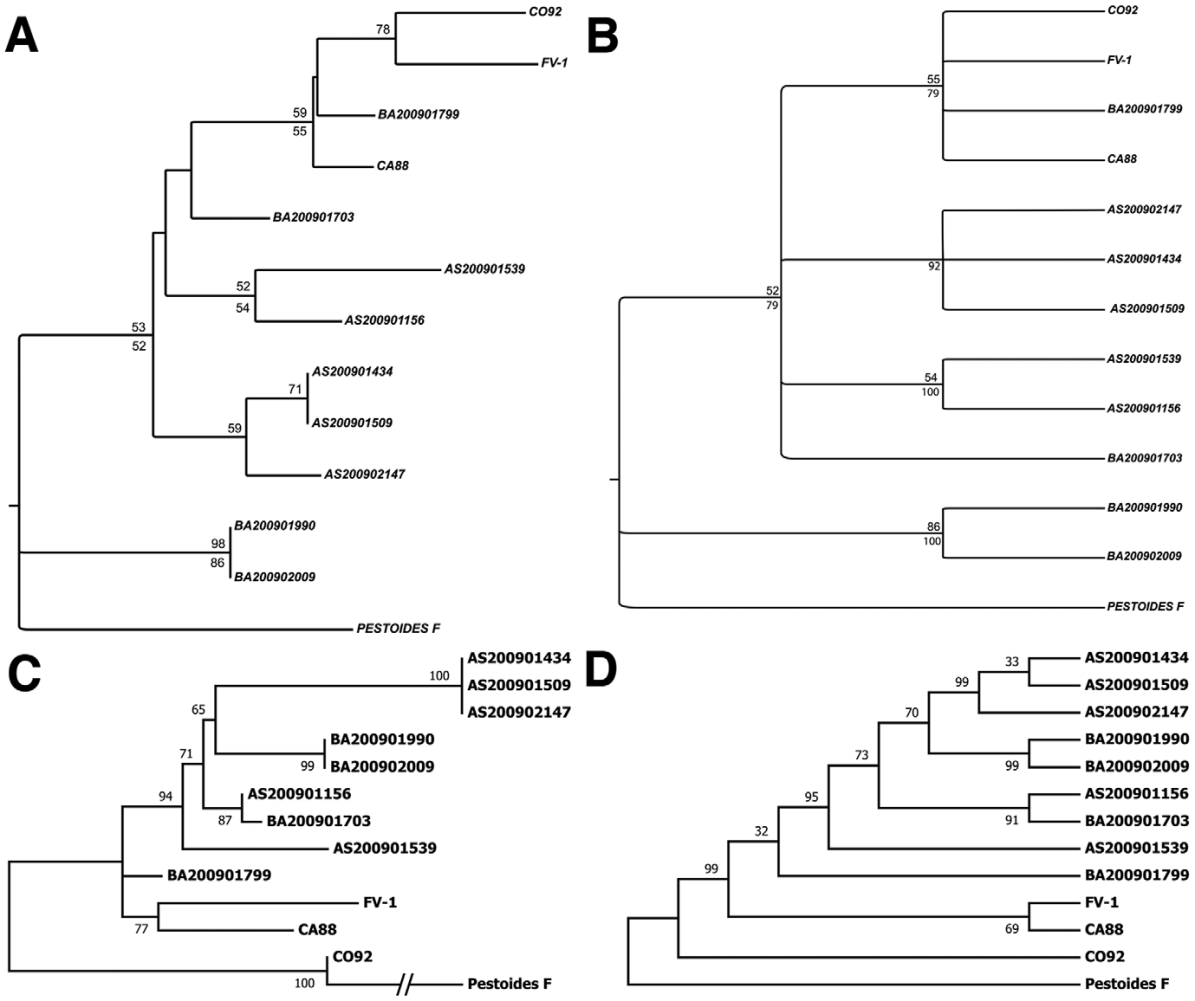
Phylogenetic Analysis of 2009 Plague Isolates. The phylogenies of the 2009 isolates were inferred from MLVA data (Panels A and B) or the SNP/Indel data (panels C and D). In all cases, the Pestoides F strain was utilized as an outgroup. **Panels A and B**: Reconstruction of relationships from MLVA data. (**A**) Neighbor Joining Analysis of MLVA data. Numbers above branches represent NJ analysis bootstrap proportions, greater than 50%, based on 1000 replications; numbers below branches represent MP analysis bootstrap support. (**B**) Maximum Parsimony analysis. Numbers above branches represent bootstrap proportions, greater than 50%, based on 1000 replications; numbers below branches represent Majority Rule consensus values. **Panels C and D**: Phylogenetic analysis using newly identified SNPs/Indels using the (**C**) Maximum Likelihood method. The tree with the highest log likelihood (−411.0996) is shown. The percentage of trees in which the associated taxa clustered together is shown next to the branches. (**D**) Phylogenetic analysis using the Maximum Parsimony method. The percentage of replicate trees in which the associated taxa clustered together in the bootstrap test (1000 replicates) are shown next to the branches. Additional details for the methods employed in phylogenetic reconstructions can be found in Materials and Methods.

**Figure 4:**
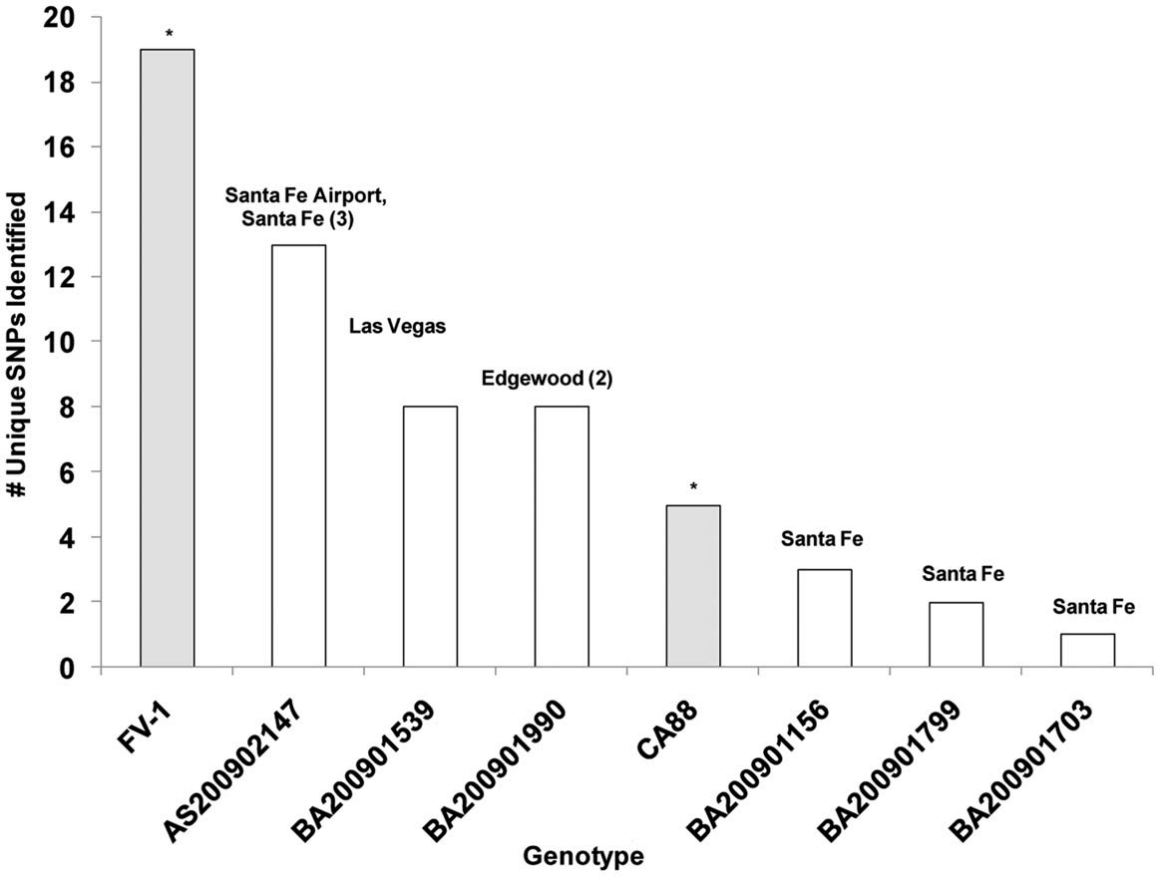
SNP Discovery in New North American *Y. pestis* Genome Sequences. The number of strain-specific newly identified SNPs relative to CO92 is plotted for each isolate. Previously sequenced strains are indicated by shaded bars with the location of origin and the number of strains showing identical genotypes in parentheses. ^*^ Number of newly identified SNPs described in references [10], [11].

Phylogenetic analyses of the MLVA and SNP/Indel data showed that unique genotypes were found in discrete geographical regions (Figure 3), and that the phylogenetic distances correlated roughly with geographic distance. For example, strain BA200901799, the strain isolated from the northernmost locale, most closely resembled CO92, whereas other strains exhibited more divergent genotypes. The strains isolated from Edgewood and Las Vegas, NM (BA200901990/2009 and AS200901539, respectively) exhibited particularly divergent genotypes with a core set of common mutations. Notably, while many new SNPs and Indels were found in this study, the number of unique SNPs for each genotype was relatively small (Figure 4).

### New IS element insertions

**Figure 5:**
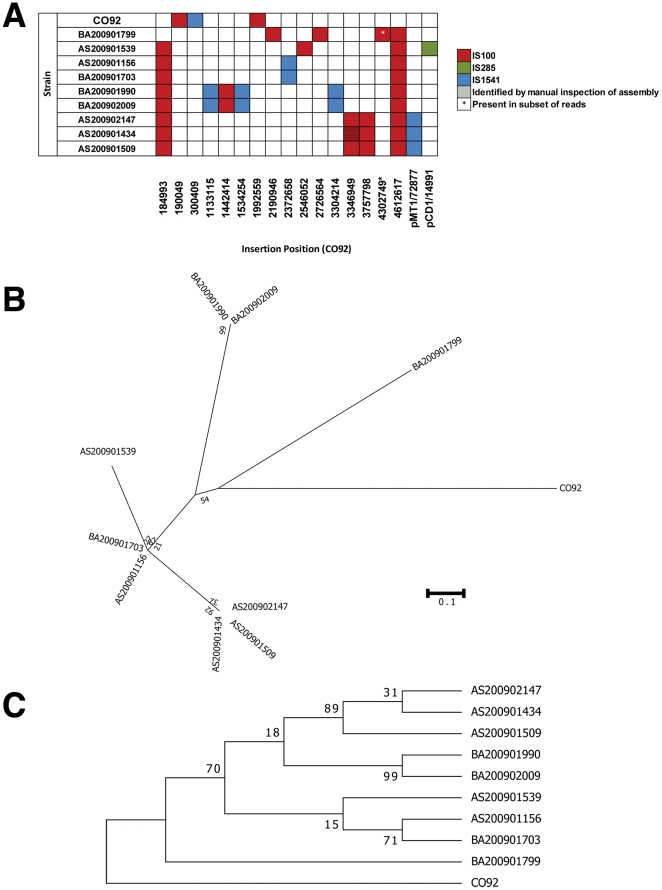
Identification of New IS Element Insertion Points in 2009 Strains. **A**) Locations of new IS element insertions. IS element insertions were identified in templated assembly experiments using CO92 as a reference. New insertion points were identified using Newbler's 454HCStructVars.txt file and the identity of the newly inserted element was determined by BLAST analysis of sequence reads containing novel junctions. Shaded square indicates the presence of an IS element beginning at the indicated nucleotide position. **B**) Phylogenetic analysis of 2009 strains using Maximum Likelihood method. Each insertion was treated as a single character. **C**) Phylogenetic analysis using Maximum Parsimony method.

Several previously unreported IS element insertion points were identified in the datasets that represent additional phylogenetic discriminators between the strains (Figure 5; Table 3). New insertion points were identified for three of the four major IS element classes in *Y. pestis*, specifically IS100, IS285, and IS1541. BLAST analysis of other *Y. pestis* strains did not reveal evidence that any of the IS element insertions identified in this study had previously been observed in other North American strains; thus the insertions identified in this study represent novel mobilization events. One of the IS100 insertion events (position 4302749), which was found in a subset of reads (Table 3; Figure S3) is predicted to inactivate the *pldA* gene encoding a putative outer membrane phospholipase A [22]. The presence of multiple variants in a WGS dataset may indicate population heterogeneity even within the single culture that generated the DNA for this sequencing project. A phylogenetic analysis of the IS element insertion pattern in these isolates supports the SNP-based analysis, but also reveals that all 9 newly-sequenced strains have a common ancestor.

**Table 3:** IS Element Variation Between Strains.

Location (CO92)	IS Element	Gene	Effect on Gene	Truncation Position (Amino Acids)
184993	IS100	YPO0166	Truncation	207/438
1133115	IS1541	Intergenic		
1442414	IS100	Intergenic		
1534254	IS1541	Intergenic		
2190946	IS100	YPO1934	Truncation	31/320
2372658	IS1541	Intergenic, near YPO2100 putative phage regulatory protein		
2546052	IS100	*fumC*/fumarate hydratase YPO2264	Truncation	275/466
2726564	IS100	*btuC*/vitamin B12 transporter YPO2425	Truncation	64/336
3304214	IS1541	Intergenic		
3346949	IS100	Putative 2-component system sensor kinase YPO2997	Truncation	411/450
3757798	IS100	*cysH (pseudogene)*	Truncation	
4302749*	IS100	*pldA* phospholipase A YPO3834	Truncation	211/293
4612617	IS100	Sugar phosphatase YPO4093	Truncation	237/270
pMT172878	IS1541	YpMT1.72c/Hypothetical protein	Truncation	88/91
pCD1 14991	IS285	YPCD1.23/Orf60 pCD1 hypothetical protein	Truncation	103/139
190049	IS100	*argD* acetylornithine aminotransferase	Intact	
300409	IS1541	Intergenic		
1992559	IS100	YPO1752	Intact	

*pldA/IS100 chimeric reads are present in a subset of 25% of reads that map to this position (See Figure S2). The remainder of the reads corresponded to an intact *pldA* gene.

## Discussion

*Yersinia pestis*, which arrived in North America during the third global pandemic in the early 20^th^ century, is commonly thought to have entered the Continental United States through the port of San Francisco [23], as some of the earliest reports of enzootic plague originated in that city. However, a recent phylogenetic analysis suggests a possible route of importation into Southern California [1]. After causing significant epidemics in the port cities of California, the organism became established in the rodent populations of the North American continent and reached its current distribution by the middle of the 20^th^ century. As a consequence of the recent introduction and the limited founder population, in contrast to the relatively diverse global population of *Y. pestis*, [1], [2], the North American population is strictly clonal in origin. Therefore, any genetic diversity in this population probably arose following its introduction.

We found that genetic diversity exists among *Y. pestis* isolates even in a restricted geographic area, and that diversification is ongoing. Our results extend the work of previous genome sequencing efforts [10], [11] which found a linear phylogeny between the CO92, CA88 and FV-1 strains of *Y. pestis*. *In silico* analysis of known SNPs places these isolates intermediate between CO92 and FV-1. By sequencing representative strains from a previously undersampled North American region [1], we found 39 additional mutations including SNPs, small indels, and changes in small tandem repeats that signify the evolution of these strains in discrete microfoci. Indeed, evolution in VNTR loci has been observed within single-season outbreaks in discrete prairie dog colonies and during *in vitro* serial transfer [20], [24]. In addition, even subtle variations in the MLVA loci correlated with the discovery of new SNPs, suggesting that analysis of MLVA profiles from new isolates may aid in the selection of more divergent strains for additional sequencing efforts. The overall concurrence of *in silico* and *in vitro* MLVA results will also allow the utilization of *in silico* MLVA as a quality-control measure on new draft assemblies, provided that read-length and coverage depth are sufficient to bridge the repeat loci. It is reasonable to expect that, over longer geographic distances, additional nucleotide level mutations would be observed. In fact, the rate of discovery of new SNPs decreased only when multiple isolates from the same geographic region were sampled (Figure 4). Only three new SNPs were shared over 8 of the 9 isolates; none were common to all.

Our dataset provides additional evidence of ongoing loss of gene function in *Y. pestis* - three of the newly identified SNPs are predicted to disrupt protein function by introducing frameshifts or nonsense mutations. Most notably, Mutation 3 (position 284552) is predicted to inactivate the HmuR hemin receptor precursor (YPO0283), which has been shown to be dispensible for virulence in a laboratory animal model [25]. The presence of such a mutation in minimally passaged isolates of *Y. pestis* constitutes further evidence that hemin uptake through the *hmu*-encoded transport system is nonessential in enzootic *Y. pestis* strains. We are currently evaluating the effects of this mutation on the ability of *Y. pestis* strains to grow on hemin as a sole iron source. Additional protein-inactivating mutations include SNPs in YPO1142 and YPO2998, which encode a putative metal transporter and a putative 2-component response-regulator, respectively. We cannot formally exclude the possibility that some of the other non-synonymous SNPs identified in these strains may also result in losses (or gains) of function. Recent enzymatic characterization by Bearden *et al.* of the AspA (aspartate-ammonia lyase) variants of enzootic strains, which contain valine at position 363 and epidemic (V363L) strains, offers a particularly compelling example in which a seemingly conservative amino acid substitution results in dramatic consequences for the activity of a given gene, and, indeed for the phenotype of the organism [26].

In addition to the potential gene functions compromised by the accumulation of SNPs, this study provides additional evidence of the ongoing activity of mobile IS elements. New insertion junctions are evident in three of the four major IS elements of *Y. pestis*. In addition, several strains have suffered insertions of IS elements into coding sequences. In one isolate, a subset of ∼25% of reads that map to this locus revealed a new insertion in the *pldA* gene encoding an outer membrane phospholipase A that would truncate the terminal 81 amino acids of the protein. The remainder of the reads at that locus mapped fully to the *pldA* locus across the putative junction. Given that *pldA* has been implicated in intravenous virulence of *Y. pseudotuberculosis* [22], the observation that this gene may be dispensable for *Y. pestis* infections in a human host would be noteworthy. However, we cannot exclude the possibility that this insertion may have occurred between the time the strain was isolated and the time DNA was prepared for sequencing. Prior to deep sequencing, detection of low levels of potential population heterogeneity would not have been possible, but the ability to do so with high-coverage datasets will contribute to forensics and attribution efforts in the future, as rare variants may not necessarily be detected by conventional plating experiments [14], [27].

Interestingly, two of the mutations identified in this study appear to have occurred in tandem. The YPO2997 and YPO2998 loci, encoding sensor kinase and response regulator components of a 2-component regulatory system, have been disrupted by an IS100 element and a nonsense mutation, respectively. While the function of these regulators remains unknown, the discovery of inactivating mutations in both genes suggests that mutating one of these components is unfavorable and that the first mutation may have predisposed the intermediate strain to accumulation of the secondary mutation. Because we did not find strains that contained only one of these mutations, it is not possible to determine the temporal order in which these occurred.

Our investigation reveals that the strains sampled in the 2009 plague season represented five distinct genotypes, four of which share a relatively recent common ancestor. Based on our phylogenetic results, we speculate that the Santa Fe region represents a nucleus of activity from which the other isolates in this study (including CO92) may have radiated. Additional sequence data from more isolates are required to confirm this hypothesis. The simultaneous re-emergence of plague strains with different genotypes in multiple locations argues for a significant role for environmental conditions in promoting the emergence of plague from a telluric lifestyle [28], [29], [30], in contrast to models that postulate the emergence of a highly virulent clone from a constantly circulating animal reservoir of relatively low virulence. Observation of a multi-genotype outbreak is also inconsistent with previously observed intentional releases of pathogens in bioterrorism incidents, in which a single dominant genotype was observed [14], [31].

Our analysis provides definitive evidence of additional molecular diversity residing within the North American plague focus. Based on the results presented here, sequencing of additional North American isolates will likely reveal considerably more genetic diversity.

## Supporting Information

Figure S1 **Representative flowgrams showing significant mutations identified in this study.**
(TIFF)

Figure S2 **Representative consensus read analysis of small Indel mutations.**
(TIFF)

Figure S3 **Identification of a representative new IS element insertion point.**
(TIFF)

Supporting Information File S1 **Sequencing and** ***de novo*** **assembly statistics, templated assembly statistics using CO92 as reference, comparison of** ***in vitro*** **and** ***in silico*** **MLVA results, and supplementary methods.**
(DOCX)

Supporting Information File S2 **Excel spreadsheet detailing High-confidence differences identified by Newbler and derived from the HCDiffs.txt files; provided as a separate file.**
(XLSX)

Supporting Information File S3 **High-confidence structural variations between CO92 and 2009 NM Isolates (Provided as separate Excel File).**
(XLSX)
