# Supplementary figures and images for: Comparative Genomics of 2009 Seasonal Plague (Yersinia pestis) in New Mexico
Source: PLoS One. 2012 Feb 16;7(2):e31604. doi: 10.1371/journal.pone.0031604 (PMC3281092; doi:10.1371/journal.pone.0031604)

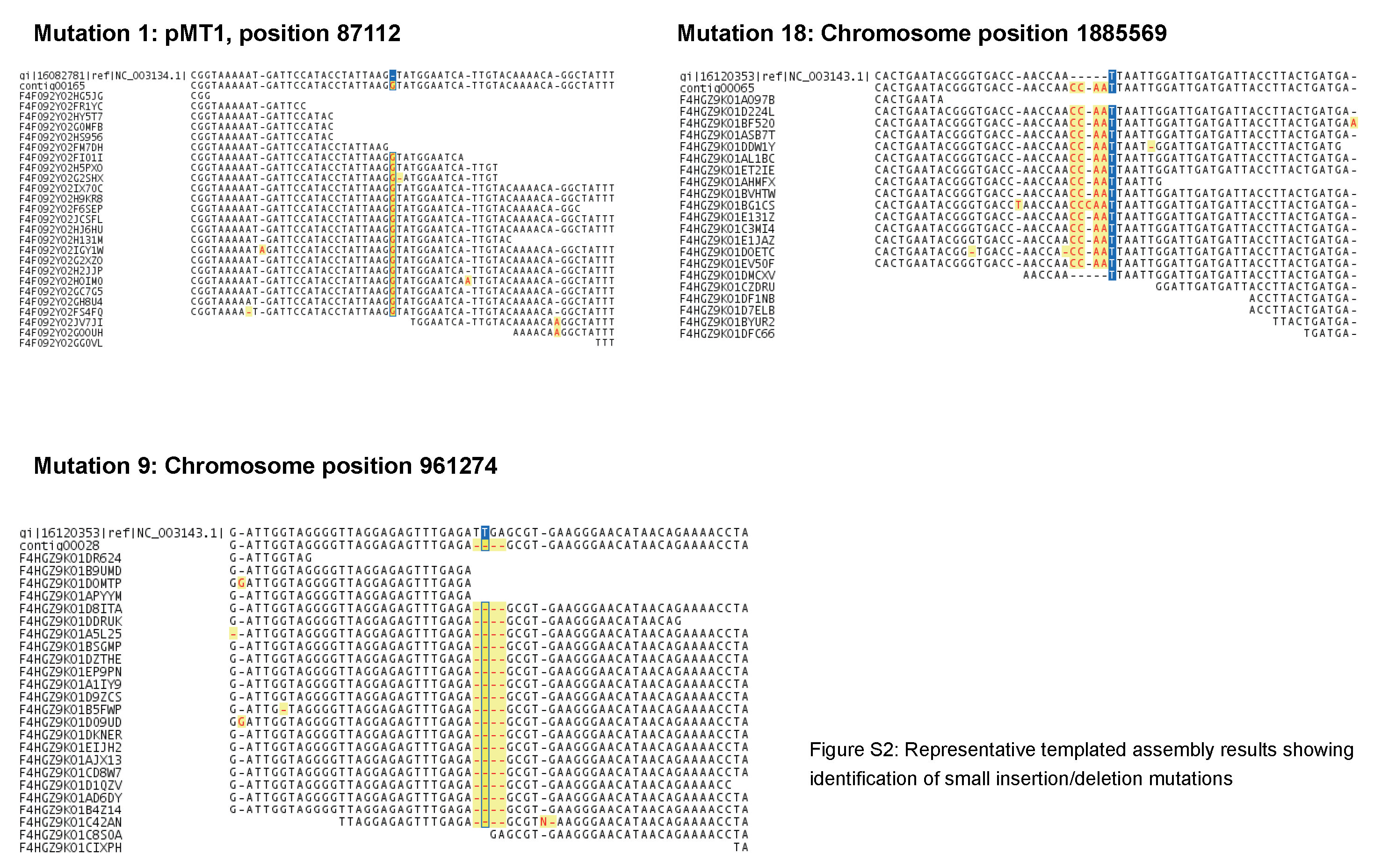

Supplement: Figure S2 — Representative consensus read analysis of small Indel mutations. (TIFF) [file pone.0031604.s002.tif]

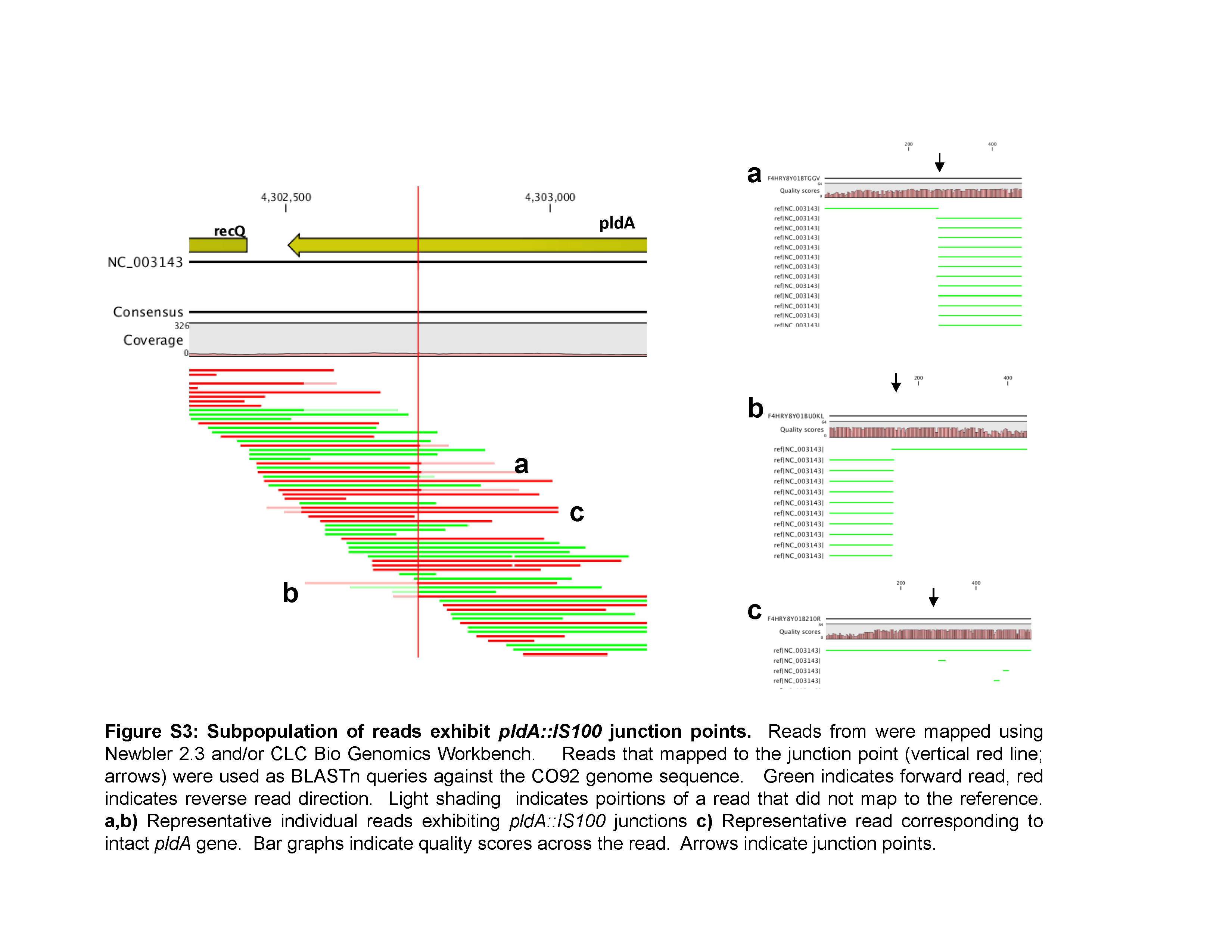

Supplement: Figure S3 — Identification of a representative new IS element insertion point. (TIFF) [file pone.0031604.s003.tif]
