# Supplementary material for: Comparative Genomics of 2009 Seasonal Plague (Yersinia pestis) in New Mexico
Source: PLoS One. 2012 Feb 16;7(2):e31604. doi: 10.1371/journal.pone.0031604 (PMC3281092; doi:10.1371/journal.pone.0031604)
Supplement: Supporting Information File S1 — Sequencing and de novo assembly statistics, templated assembly statistics using CO92 as reference, comparison of in vitro and in silico MLVA results, and supplementary methods. (DOCX) [file pone.0031604.s004.docx]

**Supplementary Data**

**Sequencing statistics and *de novo* assembly**

| **Strain Name** | **Median Depth Of Coverage** | **Number Of Large Contigs** | **N50 Contig Size** | **Largest Contig Size** | **Percent Q40 Plus Bases** | **Number Of Contigs** | **Number Of Bases** |
| --- | --- | --- | --- | --- | --- | --- | --- |
| CO92 | 38 | 180 | 41968 | 106753 | 99.99 | 207 | 4611620 |
| BA20091799 | 32 | 199 | 38104 | 104721 | 99.95 | 234 | 4597078 |
| AS200901539 | 17 | 250 | 31485 | 97549 | 99.48 | 298 | 4593061 |
| AS200901156 | 25 | 220 | 34962 | 101431 | 99.87 | 265 | 4599665 |
| BA200901703 | 15 | 282 | 27253 | 81992 | 99.5 | 345 | 4588310 |
| BA200901990 | 29 | 210 | 25993 | 106762 | 99.92 | 241 | 4539979^a^ |
| BA200902009 | 17 | 256 | 30981 | 101028 | 99.76 | 316 | 4589388 |
| AS200902147 | 15 | 255 | 30533 | 83591 | 99.37 | 466^b^ | 4641990^b^ |
| AS200901434 | 19 | 237 | 34682 | 83595 | 99.69 | 284 | 4593266 |
| AS200901509 | 18 | 222^c^ | 38963 | 104877 | 99.66^c^ | 276 | 4619754^c^ |

^a^ Smaller apparent genome size due to loss of pCD1 plasmid

^b,c^ Contamination from an ^b^unidentified *Bacillus* sp. And ^c^*Staphylococcus aureus* was detected in original sequencing data set. Templated assembly was performed to CO92 and all mapped and partially mapped reads were reassembled *de novo (*see Supplementary methods).

**Templated assembly statistics using CO92 as reference**

| **Strain ID** | **Total # Reads** | **Chromosome** | | **pCD1** | | **pMT1** | | **pPCP1** | |
| --- | --- | --- | --- | --- | --- | --- | --- | --- | --- |
|  |  | **# Reads**  **(% total)** | **Average Coverage** | **# Reads**  **(% total)** | **Average Coverage** | **# Reads**  **(% total)** | **Average Coverage** | **# Reads**  **(% total)** | **Average Coverage** |
| **CO92** | 486456 | 446282 (91.74) | 37.8 | 22739 (4.67) | 133 | 12147 (2.5) | 54.2 | 2768 (0.57) | 126 |
| **BA200901799** | 485341 | 451030 (92.93) | 32.3 | 17558 (3.62) | 86.4 | 13049 (2.4) | 49.2 | 1817 (0.37) | 70.8 |
| **AS200901539** | 399141 | 362349 (90.78) | 17.5 | 21631 (5.31) | 70.7 | 10427 (2.9) | 25.8 | 1499 (0.38) | 39.0 |
| **AS200901156** | 497592 | 449389 (90.31) | 24.8 | 30724 (6.17) | 114 | 13229 (2.66) | 38.1 | 831  (0.17) | 25.5 |
| **BA200901703** | 394370 | 362197 (91.84) | 15.8 | 14331 (3.63) | 43.5 | 12280 (3.11) | 27.4 | 1489 (0.38) | 34.3 |
| **BA200901990** | 419691 | 407081 (97.0) | 28.9 | 0 (0)^a^ | 0.71 | 10087 (2.96) | 37.5 | 594  (0.18) | 26.5 |
| **BA200902009** | 411764 | 377811 (91.75) | 16.7 | 17038 (4.14) | 50.6 | 12208 (2.96) | 27.6 | 723  (0.18) | 18.3 |
| **AS200902147** | 385590 | 352050 (91.3) | 15.26 | 11879 (3.08) | 34.6 | 10345 (2.68) | 23.15 | 1639 (0.43) | 37.4 |
| **AS200901434** | 409826 | 372218 (90.82) | 18.8 | 21545 (5.26) | 73.2 | 10727 (2.62) | 28.1 | 1824 (0.45) | 48.0 |
| **AS200901509** | 414486 | 342114 (82.54)^b^ | 19.0 | 20494 (4.94) | 76.1 | 11590 (2.8) | 32.7 | 1420 (0.34) | 42.6 |

^a^pCD1 plasmid can be lost upon *in vitro* culture ^b^Low level of apparent reads mapping to chromosome is due to sample contamination with an unspecified *Staphylococcus* spp.

**Comparison of *in vitro* and *in silico* MLVA results**

|  | **VNTR Locus^1^ Amplicon Size (bp)** | | | | | | | | | | | | | |
| --- | --- | --- | --- | --- | --- | --- | --- | --- | --- | --- | --- | --- | --- | --- |
|  | **Ms01** | | **Ms04** | | **Ms06** | | **Ms07** | | **Ms46** | | **Ms62** | | **Ms70** | |
| **Repeat unit size (bp)** | 18 | | 17 | | 60 | | 10 | | 7 | | 9 | | 9 | |
| **VNTR Method^2^** | *In vitro* | *In silico* | *In vitro* | *In silico* | *In vitro* | *In silico* | *In vitro* | *In silico* | *In vitro* | *In silico* | *In vitro* | *In silico* | *In vitro* | *In silico* |
| **CO92^3^** | 228 | 228 | 230 | 230 | 606 | 606 | 184 | 184 | 252 | 252 | 240 | 240 | 146 | 146 |
| **CO92 (reseq)** | 226 | 228 | 228 | 230 | 614 | 606 | 182 | 184 | 251 | 252 | 239 | 240 | 146 | 146 |
| **CA88** | ND | 210 | ND | 230 | ND | 606 | ND | 184 | ND | 252 | ND | 293 | ND | 146 |
| **FV-1** | ND | 192 | ND | 230 | ND | 606 | ND | 184 | ND | 252 | ND | 394 | ND | 146 |
| **Pestoides F^3^** | ND | 210 | ND | 178 | ND | 486 | ND | 193 | ND | 244 | ND | 293 | ND | 118 |
| **BA200901799** | 209 | 210 | 228 | 230 | 614 | 606 | 182 | 184 | 251 | 252 | 321 | **C** | 146 | 146 |
| **AS200901539** | 209 | 210 | 228 | 230 | 364 | 366 | 192 | 194 | 251 | 252 | 239 | 240 | 156 | 155 |
| **AS200901156** | 209 | 211 | 228 | 230 | 613 | 606 | 192 | 194 | 251 | 252 | 293 | 294 | 155 | 155 |
| **BA200901703** | 209 | 210 | 228 | 230 | 614 | 606 | 192 | 194 | 251 | 252 | 284 | 285 | 146 | 146 |
| **BA200901990** | 209 | 210 | 244 | **C**^4^ | 614 | 606 | 192 | 194 | 244 | 245 | 274 | 276 | 146 | 146 |
| **BA200902009** | 209 | 210 | 244 | **C** | 614 | **C** | 192 | 194 | 244 | 245 | 274 | 276 | 146 | 146 |
| **AS200902147** | 209 | 210 | 228 | 230 | 482 | 486 | 192 | 194 | 251 | 252 | 265 | **C** | 146 | 146 |
| **AS200901434** | 209 | 210 | 228 | 230 | 483 | 486 | 192 | 194 | 251 | 252 | 321 | **C** | 146 | 146 |
| **AS200901509** | 209 | 210 | 228 | 230 | 483 | 486 | 192 | 194 | 251 | 252 | 321 | 321 | 146 | 146 |

^1^VNTR locus designations correspond to those of Pourcel et al (2004) and Le Fleche *et al.* (2001).

^2^Loci were amplified from genomic DNA using primers published by Le Fleche *et al.* (2001). 6-FAM labeled VNTR amplicons were analyzed by capillary electrophoresis and compared to size standards. Results of *in silico* PCR using published primer sequences and the LaserGene 8.1 package are presented. Some variation from the *in vitro* results are expected due to homopolymer miscalls.

^3^*In vitro* values reported by Le Fleche *et al.* (2001). *In silico* PCR amplicon lengths are based on the complete genome sequence of *Y. pestis* CO92 from Parkhill *et al.* (2001), RefSeq accession number NC_003141.1; the Pestoides F genome by Garcia *et al.* (2007), RefSeq accession number NC_009378; and the CA88 and FV-1 draft genomes (NCBI accessions NZ_ABCD00000000 and NC_ABUD00000000, respectively). ND = not determined in this study.

^4^C denotes contig break that interrupts the VNTR locus due to insufficient read coverage and consequent repeat collapse.

**Supplementary Methods:**

**Shipment to ECBC:** Under 42 CFR Part 73, SLD is required to destroy or transfer these isolates once they are confirmed as select agents. At this time Edgewood initiates an APHIS/CDC Form 2 transfer request and SLD subcultures the isolates to TSA slants. These are incubated at 35˚C for 48 hours, and then moved to a locked 25˚C incubator until shipment. ECBC has provided SLD with labels and Category A boxes, a FedEx account number, and serves as the 24/7 Emergency contact during shipping. SLD ships the isolates once approval for APHIS/CDC Form 2 transfer request is received. Abiding by HIPAA regulations, a limited amount of information can be provided to ECBC by SLD.

**Bioinformatic Extraction of *Y. pestis* reads –** For two samples, WGS detected contaminating reads from *Bacillus* spp. or *Staphylococcus aureus.* While it is unclear at which point during the sampling, transfer, and extraction process DNA from these organisms was introduced into the samples, reads specific for *Y. pestis* were extracted from the data by mapping to the four major CO92 replicons, and extracting fully and partially mapped reads using a custom in-house Perl script. Those reads that mapped to *Y. pestis* were assembled *de novo* using Newbler.
